# 2-[4-(2-{5-*tert*-Butyl-2-chloro-3-[2-(3-pentyl-1,3-benzothia­zol-2-yl­idene)ethyl­idene]cyclo­hex-1-en­yl}ethen­yl)-3-cyano-5,5-dimethyl­furan-2-yl­idene]malono­nitrile

**DOI:** 10.1107/S1600536812050842

**Published:** 2012-12-22

**Authors:** Graeme J. Gainsford, Mohamed Ashraf, Andrew J. Kay

**Affiliations:** a Industrial Research Limited, PO Box 31-310, Lower Hutt, New Zealand

## Abstract

In the title mol­ecule, C_36_H_39_ClN_4_OS, the non-aromatic part of the cyclo­hex-1-enyl ring and the attached *tert*-butyl group are disordered over two conformations with occupancy ratios of 0.52 (3):0.48 (3) and 0.53 (3):0.47 (3), respectively. The polyene chain single- and double-bond dimensions contrast with a closely related compound [Bouit *et al.* (2007 ▶). *Chem. Mater.* **19**, 5325–5335] with an approximate 19° twist between donor and acceptor ends of the mol­ecule, related to the additional intra­molecular C—H⋯S inter­action. In the title compound, the mol­ecules pack into dimeric units about centres of symmetry utilizing weak C—H⋯N(cyano) and C—H⋯O attractive inter­actions, building both chain and ring motifs about the centres [*R*_2_^2^(8) and *R*_2_^2^(9)]. Adjacent dimeric sets then form a herringbone configuration.

## Related literature

For general background to our ongoing research involving the development of organic non-linear optical (NLO) chromophores, see: Kay *et al.* (2004 ▶); Bhuiyan *et al.* (2011 ▶). For related structures, see: Bouit *et al.* (2007 ▶, 2008 ▶); Gainsford *et al.* (2008 ▶). For a description of the Cambridge Structural Database, see: Allen (2002 ▶). For the BLA parameter, see: Marder *et al.* (1993 ▶).
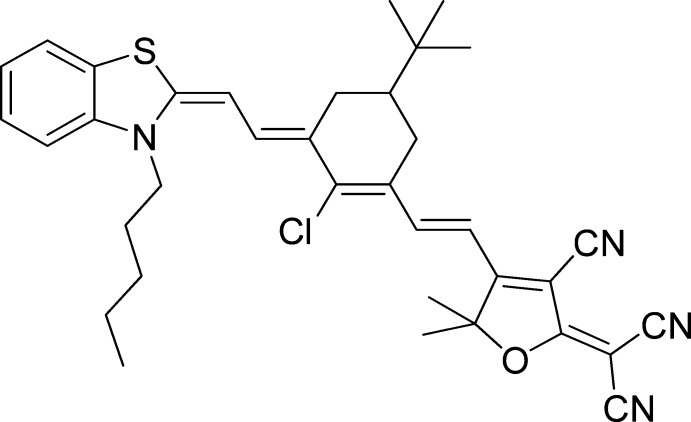


## Experimental

### 

#### Crystal data

C_36_H_39_ClN_4_OS
*M* *_r_* = 611.22
Monoclinic, 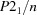

*a* = 8.6293 (5) Å
*b* = 20.1267 (11) Å
*c* = 19.5299 (11) Å
β = 102.236 (4)°
*V* = 3314.9 (3) Å^3^
*Z* = 4
Mo *K*α radiation
μ = 0.21 mm^−1^
*T* = 116 K
0.71 × 0.30 × 0.10 mm

#### Data collection

Bruker–Nonius APEXII CCD area-detector diffractometer
Absorption correction: multi-scan (Blessing, 1995 ▶) and *SADABS* (Bruker, 2005 ▶) *T*_min_ = 0.614, *T*_max_ = 0.746
32003 measured reflections
5943 independent reflections
3121 reflections with *I* > 2σ(*I*)
*R*_int_ = 0.106

#### Refinement

*R*[*F* ^2^ > 2σ(*F* ^2^)] = 0.059
*wR*(*F* ^2^) = 0.177
*S* = 1.01
5943 reflections
451 parameters
75 restraints
H atoms treated by a mixture of independent and constrained refinement
Δρ_max_ = 0.45 e Å^−3^
Δρ_min_ = −0.29 e Å^−3^

### 

Data collection: *APEX2* (Bruker, 2005 ▶); cell refinement: *SAINT* (Bruker, 2005 ▶); data reduction: *SAINT*; program(s) used to solve structure: *SHELXS97* (Sheldrick, 2008 ▶); program(s) used to refine structure: *SHELXL97* (Sheldrick, 2008 ▶); molecular graphics: *ORTEP-3* (Farrugia, 2012) ▶ and *Mercury* (Macrae *et al.*, 2008 ▶); software used to prepare material for publication: *SHELXL97* and *PLATON* (Spek, 2009 ▶).

## Supplementary Material

Crystal structure: contains datablock(s) global, I. DOI: 10.1107/S1600536812050842/bx2431sup1.cif

Structure factors: contains datablock(s) I. DOI: 10.1107/S1600536812050842/bx2431Isup2.hkl

Supplementary material file. DOI: 10.1107/S1600536812050842/bx2431Isup3.cml

Additional supplementary materials:  crystallographic information; 3D view; checkCIF report

## Figures and Tables

**Table 1:** Hydrogen-bond geometry (Å, °)

*D*—H⋯*A*	*D*—H	H⋯*A*	*D*⋯*A*	*D*—H⋯*A*
C9—H9*A*⋯O1^i^	0.98	2.59	3.494 (6)	154
C32—H32*A*⋯N2^ii^	0.98	2.73	3.702 (5)	166
C33—H33*B*⋯N1^ii^	0.98	2.65	3.539 (6)	150

Symmetry codes: (i) 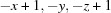; (ii) 
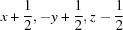
.

**Fig. 1.**
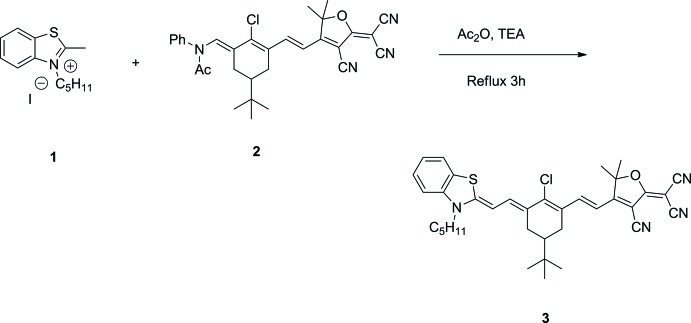
Preparation of Compound 3.

**Fig. 2.**
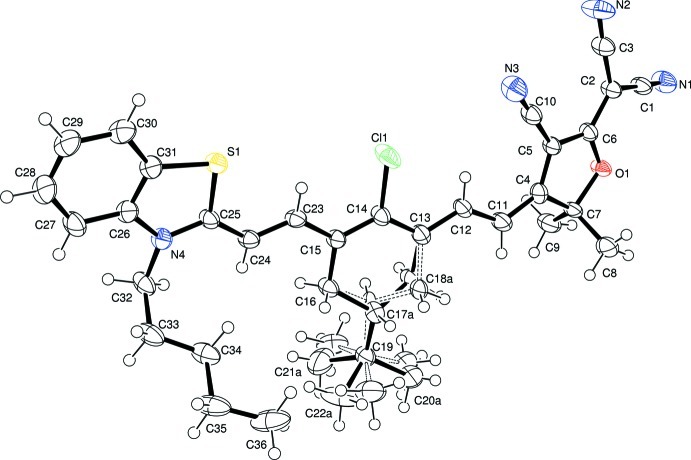
Molecular structure of the asymmetric unit (Farrugia, 1999); displacement ellipsoids are shown at the 35% probability level. The minor conformations (b) in the cyclohex-1-enyl ring and bound tert-butyl ring are shown with dotted bonds without labels to avoid confusion.

**Fig. 3.**
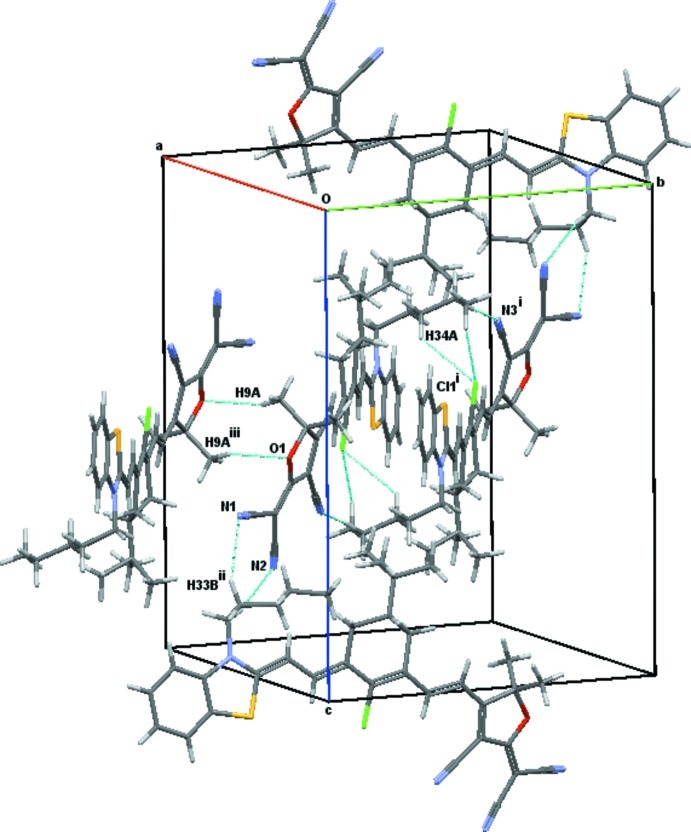
Packing diagram [Mercury, Macrae et al.,(2008)] of the unit cell. Close contacts are indicated by dotted lines. Symmetry (i) 1 - x, 1 - y, 1 - z (ii) x - 1/2, 1/2 - y, 1/2 + z (iii) 1 - x, -y, 1 - z.

## supplementary crystallographic information

### Comment

The title compound, C_36_H_39_ClN_4_OS (3, Figure 1) was synthesized as part of our ongoing research involving the development of organic nonlinear optical (NLO) chromophores. As part of this we have previously reported the crystallographic parameters for chromophores containing an indoline donor coupled to a 2-(3-cyano-4,5,5-trimethyl-5*H*-furan-2-ylidene)-malononitrile electron acceptor group (Bhuiyan *et al.*, 2011). Compound 3 was synthesized to check the impact of using a benzothiazole based donor as this should influence both the degree of both length alternation (*viz*. bond order) as well as the crystal packing. Compound 3 was conveniently prepared in good yield by the condensation of *N*-pentyl-2-methylbenzothiazolinium iodide 1 with precursor 2 (Figure 1). Compound 2 was prepared by the procedure previously reported in the literature (Kay *et al.*, 2004).

Compound REFCODES are from the C.S.D. (Version 5.33, with August 2012 updates; Allen, 2002). The asymmetric unit contents of the title compound(I) are shown in Figure 1. The 5-membered ring plane of atoms O1,C4—C7 (hereafter "CDFP", [3-cyano-5,5-dimethyl-2,5-dihydrofuran-2-ylidene]propanedinitrile) can be regarded as planar with maximum out of plane deviation for C4 of 0.029 (4) Å. The dicyano group (N1,C1,C2,C3,N2,C6) is planar but twisted by 9.4 (3) ° with respect to the "CDFP" group; this is similar to the twist in related compound NOJKUT (Gainsford *et al.*, 2008) of 5.69 (17)°, and is consistent with alleviating intramolecular contacts with the cyano group (C10–N3). The benzothiazol-2-ylidene fused ring is approximately planar with maximum out of plane distance for N4 0.026 (3) Å. This plane makes an angle of ~7° to the polyene chain atoms (C13—C16,C23,C24), which in turn is ~18° from the "CDFP" plane. These twists in the adjacent near-planar moieties contrasts with the closely related molecule HITVIQ (Bouit *et al.*,2007) where the benzothiazole entity is replaced by a 1-benzyl-3,3-dimethyl-1,3-dihydro-2*H*-indol-2-ylidene: here the CDFP and terminal donor rings make an angle of ~10°. As in HITVIQ, there are close intramolecular H···Cl interactions involving the adjacent polyene hydrogen atoms (entries 7 & 8, Table 1) but here there is an additional H···S interaction (2.68 Å, entry 9, Table 1) contributing to the twist.

The different deviation from molecular planarity is also reflected in a significant difference between the two structures in the alternation of double and single bonds beginning at the C2–C6 CDFP bond (Table 2). This alternation is described by the BLA parameter (Marder *et al.*, 1993), reflecting the average change in bond length alternation. A related sodium salt (with the CDFP ring at both ends of the molecule EGOSOJ, Bouit *et al.*, 2008)) appears to be have intermediate BLA values between the two.

The molecules pack into dimeric units about centres of symmetry utilizing weak *C*–*H*···Cyano(N) and C–H···O attractive interactions, building both chain and ring motifs about the centres (*R*_2_^2^(8) & *R*_2_^2^(9)). Table 2 summarizes those attractive interactions and key elements are shown in Figure 3. The adjacent dimeric sets then form a typical "herringbone" configuration. In contrast, the HITVIQ molecules have mainly weak, close to in-plane interactions (C–H···Cl), linked *via* chain motif weak *C*–*H*···*N*(cyano) interactions.

### Experimental

A mixture of compound 2 (5.26 g, 10 mmol) and 3-pentyl-2-methylbenzothiazolum iodide (4.51 g, 13 mmol) was stirred in the minimum amount of acetic anhydride (c. 20 ml). To this suspension, and at room temperature, was added one equivalent of triethylamine (1.4 ml, 20 mmol). The mixture was then allowed to reflux for 3 hrs, by which time its colour had changed to deep greenish black. The solvent was removed in vacuum and the residue washed with diethylether. The oily residue was then dissolved in hot isopropyl ether and kept in a fridge overnight whereupon a solid separated out. This was collected by filtration, dried under vacuum and recrystallized with hot methanol to give the title compound as a pinkish-green solid (3.66 g, 60% yield). X-Ray quality crystals were grown by slow evaporation of a solution of compound 3 in 1:1 CHCl_3_—MeOH. *M*.p. 225.8 °C. ^1^H NMR (500 MHz, CDCl_3_): δ 8.06 (d, 1H, *J* 15 Hz), 7.58 (d, 1H, *J* 10 Hz), 7.52 (d, 1H, *J* 10 Hz), 7.38 (t, 1H, *J* 4 Hz), 7.20 (t, 1H, *J* 4 Hz), 7.11 (d, 1H, *J* 8 Hz), 6.26 (d, 1H, *J* 15 Hz), 5.84 (d, 1H, *J* 12 Hz), 4.03 (t, 2H, *J* 7.2 Hz), 2.76 (t, 1H, *J* 4 Hz), 2.10 (t, 1H, *J* 4 Hz). ^13^C NMR (125 MHz, CDCl_3_): δ 175.81, 167.92, 161.02, 155.88, 153.48, 144.42, 143.37, 141.47, 135.36, 128.94, 127.57, 127.08, 123.85, 116.87, 115.97, 115.43, 114.42, 107.04, 103.02, 47.32, 42.11, 28.16, 27.95, 27.68, 27.12, 26.90, 26.25, 21.62, 13.73. LCMS Found: MNa^+^ 633.2422; C_36_H_39_ClN_4_NaOS requires MNa^+^ 633.2431; Δ = -1.4 p.p.m..

### Refinement

Nine reflections affected by the backstop and 12 others which were clearly outlier data (mostly at low angle) were omitted from the refinements (using *OMIT*). The methyl and other H atoms were refined with *U*_iso_ 1.5 & 1.2 times respectively that of the *U*_eq_ of their parent atom. All H atoms bound to carbon were constrained to their expected geometries (C—H 0.95, 0.98 & 0.99 Å).

### Crystal data

**Table:**

C_36_H_39_ClN_4_OS	*F*(000) = 1296
*M**_r_* = 611.22	*D*_x_ = 1.225 Mg m^−^^3^
Monoclinic, *P*2_1_/*n*	Mo *K*α radiation, λ = 0.71073 Å
Hall symbol: -p 2yn	Cell parameters from 3784 reflections
*a* = 8.6293 (5) Å	θ = 2.1–22.6°
*b* = 20.1267 (11) Å	µ = 0.21 mm^−^^1^
*c* = 19.5299 (11) Å	*T* = 116 K
β = 102.236 (4)°	Wedge, green
*V* = 3314.9 (3) Å^3^	0.71 × 0.30 × 0.10 mm
*Z* = 4	

### Data collection

**Table:**

Bruker–Nonius APEXII CCD area-detector diffractometer	5943 independent reflections
Radiation source: fine-focus sealed tube	3121 reflections with *I* > 2σ(*I*)
Graphite monochromator	*R*_int_ = 0.106
Detector resolution: 8.333 pixels mm^-1^	θ_max_ = 25.2°, θ_min_ = 2.9°
φ and ω scans	*h* = −10→10
Absorption correction: multi-scan (Blessing, 1995) and *SADABS* (Bruker, 2005)	*k* = 0→24
*T*_min_ = 0.614, *T*_max_ = 0.746	*l* = 0→23
32003 measured reflections	

### Refinement

**Table:**

Refinement on *F*^2^	Secondary atom site location: difference Fourier map
Least-squares matrix: full	Hydrogen site location: inferred from neighbouring sites
*R*[*F*^2^ > 2σ(*F*^2^)] = 0.059	H atoms treated by a mixture of independent and constrained refinement
*wR*(*F*^2^) = 0.177	*w* = 1/[σ^2^(*F*_o_^2^) + (0.0638*P*)^2^ + 4.4837*P*] where *P* = (*F*_o_^2^ + 2*F*_c_^2^)/3
*S* = 1.01	(Δ/σ)_max_ = 0.001
5943 reflections	Δρ_max_ = 0.45 e Å^−^^3^
451 parameters	Δρ_min_ = −0.29 e Å^−^^3^
75 restraints	Extinction correction: *SHELXL97* (Sheldrick, 2008), Fc^*^=kFc[1+0.001xFc^2^λ^3^/sin(2θ)]^-1/4^
Primary atom site location: structure-invariant direct methods	Extinction coefficient: 0.0116 (12)

### Special details

**Table:**

Geometry. All e.s.d.'s (except the e.s.d. in the dihedral angle between two l.s. planes) are estimated using the full covariance matrix. The cell e.s.d.'s are taken into account individually in the estimation of e.s.d.'s in distances, angles and torsion angles; correlations between e.s.d.'s in cell parameters are only used when they are defined by crystal symmetry. An approximate (isotropic) treatment of cell e.s.d.'s is used for estimating e.s.d.'s involving l.s. planes.
Refinement. Refinement of *F*^2^ against ALL reflections. The weighted *R*-factor *wR* and goodness of fit *S* are based on *F*^2^, conventional *R*-factors *R* are based on *F*, with *F* set to zero for negative *F*^2^. The threshold expression of *F*^2^ > σ(*F*^2^) is used only for calculating *R*-factors(gt) *etc*. and is not relevant to the choice of reflections for refinement. *R*-factors based on *F*^2^ are statistically about twice as large as those based on *F*, and *R*- factors based on ALL data will be even larger.

### Fractional atomic coordinates and isotropic or equivalent isotropic displacement parameters (Å^2^)

**Table:**

	*x*	*y*	*z*	*U*_iso_*/*U*_eq_	Occ. (<1)
S1	0.84867 (14)	0.56424 (5)	0.56589 (5)	0.0354 (3)	
Cl1	0.59822 (18)	0.34404 (6)	0.56970 (6)	0.0578 (4)	
O1	0.2567 (4)	0.01211 (13)	0.53229 (13)	0.0371 (8)	
N1	0.3033 (5)	−0.11885 (17)	0.6490 (2)	0.0515 (11)	
N2	0.4423 (6)	0.05365 (18)	0.7780 (2)	0.0587 (12)	
N3	0.4426 (5)	0.20025 (18)	0.6737 (2)	0.0510 (11)	
N4	0.8980 (4)	0.60153 (15)	0.44692 (17)	0.0339 (9)	
C1	0.3163 (5)	−0.0619 (2)	0.6503 (2)	0.0375 (11)	
C2	0.3386 (5)	0.00825 (19)	0.6541 (2)	0.0335 (10)	
C3	0.3961 (6)	0.0353 (2)	0.7218 (2)	0.0381 (11)	
C4	0.3103 (5)	0.1229 (2)	0.5049 (2)	0.0358 (11)	
C5	0.3436 (5)	0.11290 (18)	0.5781 (2)	0.0306 (10)	
C6	0.3136 (5)	0.04571 (19)	0.5921 (2)	0.0307 (10)	
C7	0.2428 (5)	0.0579 (2)	0.4718 (2)	0.0361 (11)	
C8	0.0673 (6)	0.0629 (2)	0.4383 (2)	0.0508 (13)	
H8A	0.0288	0.0197	0.4187	0.076*	
H8B	0.0518	0.0962	0.4009	0.076*	
H8C	0.0083	0.0761	0.4738	0.076*	
C9	0.3402 (6)	0.0276 (2)	0.4235 (2)	0.0473 (12)	
H9A	0.4529	0.0292	0.4462	0.071*	
H9B	0.3227	0.0528	0.3796	0.071*	
H9C	0.3079	−0.0187	0.4136	0.071*	
C10	0.3996 (5)	0.1602 (2)	0.6312 (2)	0.0359 (11)	
C11	0.3377 (6)	0.1756 (2)	0.4638 (2)	0.0444 (12)	
H11	0.2991	0.1716	0.4147	0.053*	
C12	0.4175 (5)	0.2346 (2)	0.4881 (2)	0.0359 (11)	
H12	0.4429	0.2408	0.5375	0.043*	
C13	0.4633 (5)	0.2847 (2)	0.4481 (2)	0.0372 (11)	
C14	0.5547 (5)	0.33952 (19)	0.4780 (2)	0.0320 (10)	
C15	0.6128 (5)	0.38858 (18)	0.4412 (2)	0.0294 (10)	
C16	0.5855 (6)	0.38354 (19)	0.3622 (2)	0.0331 (10)	
H16A	0.690 (2)	0.381 (2)	0.349 (2)	0.040*	
H16B	0.517 (4)	0.4212 (13)	0.3440 (19)	0.040*	
C19	0.4468 (5)	0.32423 (18)	0.2493 (2)	0.0364 (11)	
C23	0.6969 (5)	0.44466 (18)	0.4748 (2)	0.0323 (10)	
H23	0.7070	0.4486	0.5240	0.039*	
C24	0.7642 (5)	0.49323 (18)	0.4417 (2)	0.0315 (10)	
H24	0.7642	0.4882	0.3933	0.038*	
C25	0.8342 (5)	0.55100 (18)	0.4771 (2)	0.0313 (10)	
C26	0.9572 (5)	0.65384 (19)	0.4932 (2)	0.0351 (10)	
C27	1.0259 (6)	0.7125 (2)	0.4767 (2)	0.0436 (12)	
H27	1.0343	0.7220	0.4300	0.052*	
C28	1.0818 (6)	0.7565 (2)	0.5310 (3)	0.0497 (13)	
H28	1.1303	0.7967	0.5212	0.060*	
C29	1.0687 (6)	0.7433 (2)	0.5994 (3)	0.0482 (13)	
H29	1.1084	0.7745	0.6353	0.058*	
C30	0.9981 (6)	0.6849 (2)	0.6158 (2)	0.0426 (12)	
H30	0.9885	0.6757	0.6624	0.051*	
C31	0.9420 (5)	0.64050 (19)	0.5616 (2)	0.0363 (11)	
C32	0.9094 (6)	0.6037 (2)	0.3720 (2)	0.0404 (11)	
H32A	0.9185	0.5578	0.3552	0.061*	
H32B	1.0070	0.6279	0.3681	0.061*	
C33	0.7674 (6)	0.6372 (2)	0.3252 (2)	0.0476 (13)	
H33A	0.7448	0.6791	0.3478	0.071*	
H33B	0.7963	0.6491	0.2803	0.071*	
C34	0.6170 (6)	0.5956 (2)	0.3094 (2)	0.0455 (13)	
H34A	0.5902	0.5822	0.3543	0.068*	
H34B	0.6381	0.5546	0.2849	0.068*	
C35	0.4739 (7)	0.6309 (2)	0.2647 (2)	0.0566 (15)	
H35A	0.4951	0.6397	0.2177	0.085*	
H35B	0.4586	0.6741	0.2865	0.085*	
C36	0.3223 (7)	0.5897 (3)	0.2571 (3)	0.0679 (18)	
H36A	0.3330	0.5488	0.2312	0.102*	
H36B	0.2321	0.6155	0.2315	0.102*	
H36C	0.3046	0.5784	0.3036	0.102*	
C17A	0.456 (2)	0.3354 (9)	0.3291 (9)	0.030 (3)	0.52 (3)
H17B	0.3546	0.3578	0.3321	0.045*	0.52 (3)
C18A	0.457 (3)	0.2721 (7)	0.3690 (5)	0.041 (4)	0.52 (3)
H18A	0.5507	0.2453	0.3641	0.061*	0.52 (3)
H18B	0.3610	0.2462	0.3488	0.061*	0.52 (3)
C20A	0.327 (3)	0.2697 (10)	0.2179 (17)	0.065 (7)	0.53 (3)
H20D	0.2282	0.2761	0.2341	0.097*	0.53 (3)
H20E	0.3716	0.2260	0.2331	0.097*	0.53 (3)
H20F	0.3059	0.2723	0.1667	0.097*	0.53 (3)
C21A	0.5878 (18)	0.3349 (16)	0.2147 (10)	0.082 (7)	0.53 (3)
H21D	0.5520	0.3323	0.1637	0.123*	0.53 (3)
H21E	0.6678	0.3006	0.2307	0.123*	0.53 (3)
H21F	0.6342	0.3788	0.2276	0.123*	0.53 (3)
C22A	0.389 (4)	0.3895 (7)	0.2107 (8)	0.076 (7)	0.53 (3)
H22D	0.2902	0.4036	0.2232	0.113*	0.53 (3)
H22E	0.3711	0.3823	0.1600	0.113*	0.53 (3)
H22F	0.4700	0.4240	0.2244	0.113*	0.53 (3)
C17B	0.515 (2)	0.3180 (8)	0.3303 (9)	0.023 (3)	0.48 (3)
H17A	0.6009	0.2839	0.3379	0.035*	0.48 (3)
C18B	0.385 (2)	0.2951 (8)	0.3710 (5)	0.034 (4)	0.48 (3)
H18C	0.3012	0.3293	0.3667	0.051*	0.48 (3)
H18D	0.3357	0.2531	0.3507	0.051*	0.48 (3)
C20B	0.387 (3)	0.2544 (7)	0.225 (2)	0.055 (6)	0.47 (3)
H20A	0.2956	0.2430	0.2455	0.083*	0.47 (3)
H20B	0.4715	0.2219	0.2402	0.083*	0.47 (3)
H20C	0.3541	0.2537	0.1738	0.083*	0.47 (3)
C21B	0.6072 (14)	0.3068 (11)	0.2329 (10)	0.059 (5)	0.47 (3)
H21A	0.5937	0.2972	0.1828	0.089*	0.47 (3)
H21B	0.6509	0.2676	0.2601	0.089*	0.47 (3)
H21C	0.6801	0.3443	0.2454	0.089*	0.47 (3)
C22B	0.3139 (19)	0.3755 (8)	0.2311 (13)	0.063 (5)	0.47 (3)
H22A	0.2369	0.3684	0.2607	0.095*	0.47 (3)
H22B	0.2610	0.3707	0.1817	0.095*	0.47 (3)
H22C	0.3586	0.4203	0.2391	0.095*	0.47 (3)

### Atomic displacement parameters (Å^2^)

**Table:**

	*U*^11^	*U*^22^	*U*^33^	*U*^12^	*U*^13^	*U*^23^
S1	0.0532 (8)	0.0223 (5)	0.0285 (6)	−0.0038 (5)	0.0034 (5)	−0.0020 (4)
Cl1	0.1076 (12)	0.0406 (6)	0.0267 (6)	−0.0273 (7)	0.0177 (6)	−0.0036 (5)
O1	0.058 (2)	0.0241 (14)	0.0249 (15)	−0.0138 (14)	0.0002 (14)	0.0032 (12)
N1	0.079 (3)	0.027 (2)	0.045 (2)	−0.009 (2)	0.006 (2)	0.0054 (17)
N2	0.105 (4)	0.035 (2)	0.029 (2)	0.005 (2)	−0.001 (2)	0.0003 (18)
N3	0.077 (3)	0.030 (2)	0.046 (2)	−0.008 (2)	0.014 (2)	−0.0047 (18)
N4	0.047 (2)	0.0196 (16)	0.033 (2)	−0.0038 (16)	0.0037 (17)	−0.0035 (14)
C1	0.053 (3)	0.028 (2)	0.028 (2)	−0.006 (2)	0.002 (2)	0.0060 (18)
C2	0.050 (3)	0.022 (2)	0.027 (2)	−0.0055 (19)	0.005 (2)	−0.0002 (16)
C3	0.055 (3)	0.024 (2)	0.033 (3)	0.002 (2)	0.006 (2)	0.0050 (19)
C4	0.041 (3)	0.028 (2)	0.037 (3)	−0.010 (2)	0.007 (2)	0.0028 (19)
C5	0.042 (3)	0.0221 (19)	0.026 (2)	−0.0084 (19)	0.0049 (19)	0.0000 (16)
C6	0.040 (3)	0.025 (2)	0.027 (2)	−0.0045 (19)	0.0082 (19)	0.0009 (17)
C7	0.051 (3)	0.029 (2)	0.023 (2)	−0.014 (2)	−0.002 (2)	0.0077 (17)
C8	0.062 (3)	0.040 (3)	0.044 (3)	−0.016 (3)	−0.003 (2)	0.014 (2)
C9	0.068 (4)	0.042 (3)	0.030 (2)	−0.008 (2)	0.005 (2)	0.003 (2)
C10	0.050 (3)	0.025 (2)	0.034 (2)	−0.003 (2)	0.009 (2)	0.0043 (19)
C11	0.061 (3)	0.037 (2)	0.031 (2)	−0.018 (2)	0.000 (2)	0.0072 (19)
C12	0.046 (3)	0.031 (2)	0.030 (2)	−0.009 (2)	0.005 (2)	0.0071 (18)
C13	0.047 (3)	0.033 (2)	0.030 (2)	−0.011 (2)	0.006 (2)	0.0041 (18)
C14	0.044 (3)	0.024 (2)	0.029 (2)	−0.004 (2)	0.010 (2)	0.0002 (17)
C15	0.041 (3)	0.0191 (19)	0.027 (2)	−0.0022 (18)	0.0034 (19)	−0.0011 (16)
C16	0.049 (3)	0.023 (2)	0.028 (2)	−0.005 (2)	0.008 (2)	−0.0001 (17)
C19	0.049 (3)	0.029 (2)	0.030 (2)	−0.007 (2)	0.007 (2)	−0.0017 (18)
C23	0.045 (3)	0.024 (2)	0.025 (2)	−0.0021 (19)	0.0005 (19)	−0.0014 (16)
C24	0.042 (3)	0.025 (2)	0.025 (2)	0.0014 (19)	0.0030 (19)	−0.0003 (17)
C25	0.042 (3)	0.022 (2)	0.029 (2)	−0.0008 (19)	0.005 (2)	0.0027 (16)
C26	0.046 (3)	0.020 (2)	0.037 (2)	−0.0007 (19)	0.001 (2)	−0.0027 (18)
C27	0.060 (3)	0.025 (2)	0.045 (3)	−0.002 (2)	0.010 (2)	0.007 (2)
C28	0.063 (4)	0.020 (2)	0.062 (3)	−0.004 (2)	0.003 (3)	−0.002 (2)
C29	0.057 (3)	0.024 (2)	0.055 (3)	−0.001 (2)	−0.006 (3)	−0.007 (2)
C30	0.053 (3)	0.032 (2)	0.040 (3)	0.003 (2)	0.001 (2)	−0.008 (2)
C31	0.046 (3)	0.0175 (19)	0.041 (3)	0.0009 (19)	0.001 (2)	−0.0007 (18)
C32	0.067 (3)	0.022 (2)	0.033 (2)	−0.005 (2)	0.015 (2)	0.0000 (18)
C33	0.085 (4)	0.026 (2)	0.031 (2)	0.001 (2)	0.010 (3)	0.0016 (19)
C34	0.076 (4)	0.026 (2)	0.033 (3)	0.009 (2)	0.007 (2)	−0.0007 (19)
C35	0.096 (5)	0.037 (3)	0.029 (3)	0.017 (3)	−0.005 (3)	−0.005 (2)
C36	0.093 (5)	0.050 (3)	0.044 (3)	0.018 (3)	−0.024 (3)	−0.016 (2)
C17A	0.042 (10)	0.023 (7)	0.028 (5)	−0.002 (6)	0.015 (7)	−0.007 (5)
C18A	0.063 (10)	0.024 (6)	0.032 (5)	−0.011 (6)	0.003 (5)	0.005 (4)
C20A	0.093 (17)	0.066 (10)	0.028 (7)	−0.042 (11)	−0.006 (13)	−0.009 (9)
C21A	0.063 (10)	0.14 (2)	0.042 (9)	−0.036 (10)	0.011 (7)	−0.008 (10)
C22A	0.127 (19)	0.057 (8)	0.033 (7)	0.043 (10)	−0.005 (9)	0.002 (5)
C17B	0.028 (9)	0.014 (7)	0.030 (5)	−0.006 (5)	0.012 (6)	−0.011 (5)
C18B	0.044 (9)	0.022 (6)	0.034 (5)	−0.012 (5)	0.004 (5)	0.000 (4)
C20B	0.072 (15)	0.050 (9)	0.040 (13)	−0.017 (9)	0.004 (12)	−0.015 (9)
C21B	0.079 (11)	0.073 (12)	0.019 (8)	0.002 (8)	−0.001 (6)	−0.021 (7)
C22B	0.079 (12)	0.051 (8)	0.049 (11)	0.001 (8)	−0.013 (7)	0.004 (7)

### Geometric parameters (Å, º)

**Table:**

S1—C25	1.732 (4)	C24—H24	0.9500
S1—C31	1.743 (4)	C26—C27	1.389 (6)
Cl1—C14	1.752 (4)	C26—C31	1.394 (6)
O1—C6	1.349 (4)	C27—C28	1.388 (6)
O1—C7	1.482 (4)	C27—H27	0.9500
N1—C1	1.151 (5)	C28—C29	1.389 (6)
N2—C3	1.147 (5)	C28—H28	0.9500
N3—C10	1.160 (5)	C29—C30	1.393 (6)
N4—C25	1.350 (5)	C29—H29	0.9500
N4—C26	1.411 (5)	C30—C31	1.392 (6)
N4—C32	1.487 (5)	C30—H30	0.9500
C1—C2	1.426 (5)	C32—C33	1.523 (6)
C2—C6	1.404 (5)	C32—H32A	0.9900
C2—C3	1.418 (6)	C32—H32B	0.9900
C4—C11	1.380 (5)	C33—C34	1.521 (6)
C4—C5	1.412 (5)	C33—H33A	0.9900
C4—C7	1.519 (5)	C33—H33B	0.9900
C5—C6	1.414 (5)	C34—C35	1.528 (6)
C5—C10	1.415 (6)	C34—H34A	0.9900
C7—C9	1.517 (6)	C34—H34B	0.9900
C7—C8	1.520 (6)	C35—C36	1.529 (7)
C8—H8A	0.9800	C35—H35A	0.9900
C8—H8B	0.9800	C35—H35B	0.9900
C8—H8C	0.9800	C36—H36A	0.9800
C9—H9A	0.9800	C36—H36B	0.9800
C9—H9B	0.9800	C36—H36C	0.9800
C9—H9C	0.9800	C17A—C18A	1.49 (3)
C11—C12	1.404 (6)	C17A—H17B	1.0000
C11—H11	0.9500	C18A—H18A	0.9900
C12—C13	1.384 (5)	C18A—H18B	0.9900
C12—H12	0.9500	C20A—H20D	0.9800
C13—C14	1.410 (5)	C20A—H20E	0.9800
C13—C18B	1.529 (10)	C20A—H20F	0.9800
C13—C18A	1.556 (10)	C21A—H21D	0.9800
C14—C15	1.377 (5)	C21A—H21E	0.9800
C15—C23	1.424 (5)	C21A—H21F	0.9800
C15—C16	1.513 (5)	C22A—H22D	0.9800
C16—C17A	1.515 (17)	C22A—H22E	0.9800
C16—C17B	1.530 (15)	C22A—H22F	0.9800
C16—H16A	0.992 (10)	C17B—C18B	1.57 (3)
C16—H16B	0.982 (10)	C17B—H17A	1.0000
C19—C21B	1.526 (8)	C18B—H18C	0.9900
C19—C21A	1.527 (8)	C18B—H18D	0.9900
C19—C22B	1.528 (8)	C20B—H20A	0.9800
C19—C20B	1.539 (8)	C20B—H20B	0.9800
C19—C20A	1.542 (8)	C20B—H20C	0.9800
C19—C22A	1.544 (7)	C21B—H21A	0.9800
C19—C17A	1.559 (17)	C21B—H21B	0.9800
C19—C17B	1.571 (18)	C21B—H21C	0.9800
C23—C24	1.367 (5)	C22B—H22A	0.9800
C23—H23	0.9500	C22B—H22B	0.9800
C24—C25	1.421 (5)	C22B—H22C	0.9800
			
C25—S1—C31	91.3 (2)	C27—C28—H28	119.1
C6—O1—C7	109.4 (3)	C29—C28—H28	119.1
C25—N4—C26	114.1 (3)	C28—C29—C30	120.7 (4)
C25—N4—C32	124.8 (3)	C28—C29—H29	119.6
C26—N4—C32	121.2 (3)	C30—C29—H29	119.6
N1—C1—C2	177.6 (5)	C31—C30—C29	117.8 (4)
C6—C2—C3	123.9 (3)	C31—C30—H30	121.1
C6—C2—C1	119.5 (4)	C29—C30—H30	121.1
C3—C2—C1	116.4 (3)	C30—C31—C26	121.1 (4)
N2—C3—C2	176.2 (4)	C30—C31—S1	128.2 (4)
C11—C4—C5	132.4 (4)	C26—C31—S1	110.7 (3)
C11—C4—C7	120.8 (4)	N4—C32—C33	113.1 (4)
C5—C4—C7	106.6 (3)	N4—C32—H32A	109.0
C4—C5—C6	108.9 (3)	C33—C32—H32A	109.0
C4—C5—C10	127.6 (4)	N4—C32—H32B	109.0
C6—C5—C10	123.4 (4)	C33—C32—H32B	109.0
O1—C6—C2	116.0 (3)	H32A—C32—H32B	107.8
O1—C6—C5	111.1 (3)	C34—C33—C32	114.9 (3)
C2—C6—C5	132.8 (4)	C34—C33—H33A	108.6
O1—C7—C9	106.5 (3)	C32—C33—H33A	108.6
O1—C7—C4	103.7 (3)	C34—C33—H33B	108.6
C9—C7—C4	113.3 (4)	C32—C33—H33B	108.6
O1—C7—C8	106.8 (3)	H33A—C33—H33B	107.5
C9—C7—C8	113.1 (4)	C33—C34—C35	114.3 (4)
C4—C7—C8	112.5 (4)	C33—C34—H34A	108.7
C7—C8—H8A	109.5	C35—C34—H34A	108.7
C7—C8—H8B	109.5	C33—C34—H34B	108.7
H8A—C8—H8B	109.5	C35—C34—H34B	108.7
C7—C8—H8C	109.5	H34A—C34—H34B	107.6
H8A—C8—H8C	109.5	C34—C35—C36	112.0 (4)
H8B—C8—H8C	109.5	C34—C35—H35A	109.2
C7—C9—H9A	109.5	C36—C35—H35A	109.2
C7—C9—H9B	109.5	C34—C35—H35B	109.2
H9A—C9—H9B	109.5	C36—C35—H35B	109.2
C7—C9—H9C	109.5	H35A—C35—H35B	107.9
H9A—C9—H9C	109.5	C35—C36—H36A	109.5
H9B—C9—H9C	109.5	C35—C36—H36B	109.5
N3—C10—C5	178.1 (5)	H36A—C36—H36B	109.5
C4—C11—C12	125.8 (4)	C35—C36—H36C	109.5
C4—C11—H11	117.1	H36A—C36—H36C	109.5
C12—C11—H11	117.1	H36B—C36—H36C	109.5
C13—C12—C11	127.1 (4)	C18A—C17A—C16	113.6 (15)
C13—C12—H12	116.4	C18A—C17A—C19	113.1 (13)
C11—C12—H12	116.4	C16—C17A—C19	113.4 (11)
C12—C13—C14	122.6 (4)	C18A—C17A—H17B	105.2
C12—C13—C18B	122.5 (6)	C16—C17A—H17B	105.2
C14—C13—C18B	113.1 (6)	C19—C17A—H17B	105.2
C12—C13—C18A	119.3 (5)	C17A—C18A—C13	112.0 (13)
C14—C13—C18A	115.9 (5)	C17A—C18A—H18A	109.2
C15—C14—C13	125.3 (4)	C13—C18A—H18A	109.2
C15—C14—Cl1	118.3 (3)	C17A—C18A—H18B	109.2
C13—C14—Cl1	116.4 (3)	C13—C18A—H18B	109.2
C14—C15—C23	122.3 (4)	H18A—C18A—H18B	107.9
C14—C15—C16	119.3 (3)	C19—C20A—H20D	109.5
C23—C15—C16	118.5 (3)	C19—C20A—H20E	109.5
C15—C16—C17A	114.8 (7)	C19—C20A—H20F	109.5
C15—C16—C17B	115.8 (7)	C19—C21A—H21D	109.5
C15—C16—H16A	109 (2)	C19—C21A—H21E	109.5
C17A—C16—H16A	119 (3)	C19—C21A—H21F	109.5
C15—C16—H16B	106 (2)	C19—C22A—H22D	109.5
C17B—C16—H16B	111 (3)	C19—C22A—H22E	109.5
C21B—C19—C22B	140.9 (18)	C19—C22A—H22F	109.5
C21B—C19—C20B	89.3 (17)	C16—C17B—C18B	108.3 (14)
C22B—C19—C20B	110.9 (12)	C16—C17B—C19	111.9 (10)
C21B—C19—C20A	108.2 (17)	C18B—C17B—C19	112.3 (13)
C21A—C19—C20A	116.9 (18)	C16—C17B—H17A	108.1
C22B—C19—C20A	89.0 (12)	C18B—C17B—H17A	108.1
C21B—C19—C22A	107.8 (19)	C19—C17B—H17A	108.1
C21A—C19—C22A	82 (2)	C13—C18B—C17B	109.0 (13)
C20A—C19—C22A	107.0 (12)	C13—C18B—H18C	109.9
C21A—C19—C17A	123.0 (11)	C17B—C18B—H18C	109.9
C20A—C19—C17A	112.9 (15)	C13—C18B—H18D	109.9
C22A—C19—C17A	108.0 (10)	C17B—C18B—H18D	109.9
C21B—C19—C17B	91.9 (11)	H18C—C18B—H18D	108.3
C22B—C19—C17B	112.9 (12)	C19—C20B—H20A	109.5
C20B—C19—C17B	105.5 (16)	C19—C20B—H20B	109.5
C24—C23—C15	125.1 (4)	H20A—C20B—H20B	109.5
C24—C23—H23	117.4	C19—C20B—H20C	109.5
C15—C23—H23	117.4	H20A—C20B—H20C	109.5
C23—C24—C25	122.3 (4)	H20B—C20B—H20C	109.5
C23—C24—H24	118.8	C19—C21B—H21A	109.5
C25—C24—H24	118.8	C19—C21B—H21B	109.5
N4—C25—C24	125.0 (4)	H21A—C21B—H21B	109.5
N4—C25—S1	111.8 (3)	C19—C21B—H21C	109.5
C24—C25—S1	123.1 (3)	H21A—C21B—H21C	109.5
C27—C26—C31	121.2 (4)	H21B—C21B—H21C	109.5
C27—C26—N4	126.8 (4)	C19—C22B—H22A	109.5
C31—C26—N4	112.0 (3)	C19—C22B—H22B	109.5
C28—C27—C26	117.4 (4)	H22A—C22B—H22B	109.5
C28—C27—H27	121.3	C19—C22B—H22C	109.5
C26—C27—H27	121.3	H22A—C22B—H22C	109.5
C27—C28—C29	121.8 (4)	H22B—C22B—H22C	109.5
			
C11—C4—C5—C6	−170.6 (5)	C23—C24—C25—S1	2.7 (6)
C7—C4—C5—C6	4.6 (5)	C31—S1—C25—N4	0.5 (3)
C11—C4—C5—C10	8.7 (9)	C31—S1—C25—C24	−180.0 (4)
C7—C4—C5—C10	−176.1 (4)	C25—N4—C26—C27	−178.6 (4)
C7—O1—C6—C2	−177.9 (4)	C32—N4—C26—C27	2.2 (7)
C7—O1—C6—C5	−0.8 (5)	C25—N4—C26—C31	2.3 (5)
C3—C2—C6—O1	−179.3 (4)	C32—N4—C26—C31	−177.0 (4)
C1—C2—C6—O1	5.2 (6)	C31—C26—C27—C28	1.4 (7)
C3—C2—C6—C5	4.4 (8)	N4—C26—C27—C28	−177.7 (4)
C1—C2—C6—C5	−171.1 (5)	C26—C27—C28—C29	−0.7 (7)
C4—C5—C6—O1	−2.5 (5)	C27—C28—C29—C30	−0.1 (8)
C10—C5—C6—O1	178.1 (4)	C28—C29—C30—C31	0.2 (7)
C4—C5—C6—C2	174.0 (5)	C29—C30—C31—C26	0.5 (7)
C10—C5—C6—C2	−5.4 (8)	C29—C30—C31—S1	−179.8 (4)
C6—O1—C7—C9	123.3 (4)	C27—C26—C31—C30	−1.4 (7)
C6—O1—C7—C4	3.5 (4)	N4—C26—C31—C30	177.9 (4)
C6—O1—C7—C8	−115.6 (4)	C27—C26—C31—S1	179.0 (4)
C11—C4—C7—O1	171.0 (4)	N4—C26—C31—S1	−1.8 (5)
C5—C4—C7—O1	−4.8 (5)	C25—S1—C31—C30	−178.9 (4)
C11—C4—C7—C9	55.9 (6)	C25—S1—C31—C26	0.8 (3)
C5—C4—C7—C9	−119.9 (4)	C25—N4—C32—C33	91.7 (5)
C11—C4—C7—C8	−73.9 (5)	C26—N4—C32—C33	−89.1 (5)
C5—C4—C7—C8	110.3 (4)	N4—C32—C33—C34	−75.2 (5)
C5—C4—C11—C12	3.2 (9)	C32—C33—C34—C35	177.7 (4)
C7—C4—C11—C12	−171.5 (4)	C33—C34—C35—C36	−173.9 (4)
C4—C11—C12—C13	171.7 (5)	C15—C16—C17A—C18A	−41 (2)
C11—C12—C13—C14	−174.6 (5)	C15—C16—C17A—C19	−172.2 (9)
C11—C12—C13—C18B	21.9 (12)	C21A—C19—C17A—C18A	−107 (2)
C11—C12—C13—C18A	−12.4 (12)	C20A—C19—C17A—C18A	43 (3)
C12—C13—C14—C15	175.5 (4)	C22A—C19—C17A—C18A	161 (2)
C18B—C13—C14—C15	−19.7 (11)	C21A—C19—C17A—C16	25 (2)
C18A—C13—C14—C15	12.7 (11)	C20A—C19—C17A—C16	173.9 (16)
C12—C13—C14—Cl1	−3.8 (6)	C22A—C19—C17A—C16	−68 (2)
C18B—C13—C14—Cl1	161.1 (9)	C16—C17A—C18A—C13	50 (3)
C18A—C13—C14—Cl1	−166.6 (10)	C19—C17A—C18A—C13	−178.8 (8)
C13—C14—C15—C23	176.4 (4)	C12—C13—C18A—C17A	160.6 (15)
Cl1—C14—C15—C23	−4.4 (6)	C14—C13—C18A—C17A	−36 (2)
C13—C14—C15—C16	−3.0 (7)	C15—C16—C17B—C18B	39.7 (18)
Cl1—C14—C15—C16	176.2 (3)	C17A—C16—C17B—C18B	−53 (3)
C14—C15—C16—C17A	17.0 (11)	C15—C16—C17B—C19	164.0 (8)
C23—C15—C16—C17A	−162.4 (10)	C21B—C19—C17B—C16	88.1 (15)
C14—C15—C16—C17B	−8.5 (10)	C22B—C19—C17B—C16	−60.8 (17)
C23—C15—C16—C17B	172.1 (9)	C20B—C19—C17B—C16	177.9 (16)
C14—C15—C23—C24	176.6 (4)	C21B—C19—C17B—C18B	−149.9 (17)
C16—C15—C23—C24	−4.1 (6)	C22B—C19—C17B—C18B	61.1 (19)
C15—C23—C24—C25	174.2 (4)	C20B—C19—C17B—C18B	−60 (2)
C26—N4—C25—C24	178.8 (4)	C12—C13—C18B—C17B	−143.8 (11)
C32—N4—C25—C24	−2.0 (7)	C14—C13—C18B—C17B	51.3 (18)
C26—N4—C25—S1	−1.6 (5)	C16—C17B—C18B—C13	−61 (2)
C32—N4—C25—S1	177.6 (3)	C19—C17B—C18B—C13	175.3 (7)
C23—C24—C25—N4	−177.8 (4)		

### Hydrogen-bond geometry (Å, º)

**Table:**

*D*—H···*A*	*D*—H	H···*A*	*D*···*A*	*D*—H···*A*
C9—H9*A*···O1^i^	0.98	2.59	3.494 (6)	154
C32—H32*A*···N2^ii^	0.98	2.73	3.702 (5)	166
C33—H33*B*···N1^ii^	0.98	2.65	3.539 (6)	150

Symmetry codes: (i) −*x*+1, −*y*, −*z*+1; (ii) *x*+1/2, −*y*+1/2, *z*−1/2.

### Bond Distances(Å)^a^

**Table:**

AtomA—AtomB	Here	HITVIQ	EGOSOJ
C2–C6	1.404 (5)	1.378 (3)	1.385 (6)
C6–C7	1.414 (5)	1.429 (3)	1.417 (6)
C7–C4	1.412 (5)	1.370 (3)	1.402 (6)
C4–c11	1.381 (6)	1.416 (3)	1.373 (5)
Average db^a^	1.411 (9)	1.368 (7)	1.397 (8)
Average sb^a^	1.385 (18)	1.426 (10)	1.395 (16)
BLA^b^	-0.026	0.058	-0.002

a. db,sb double,single bonds (10). b. Marder *et al.*, 1993.
